# Novel *WT1* Missense Mutations in Han Chinese Women with Premature Ovarian Failure

**DOI:** 10.1038/srep13983

**Published:** 2015-09-11

**Authors:** Huidan Wang, Guangyu Li, Jun Zhang, Fei Gao, Weiping Li, Yingying Qin, Zi-Jiang Chen

**Affiliations:** 1 Center for Reproductive Medicine, Provincial Hospital Affiliated to Shandong University, National Research Center for Assisted Reproductive Technology and Reproductive Genetics, The Key laboratory for Reproductive Endocrinology of Ministry of Education, Shandong Provincial Key Laboratory of Reproductive Medicine, Jinan, Shandong, China; 2 Institute of Zoology, Chinese Academy of Sciences, State Key Laboratory of Reproduction Biology, Beijing, China; 3 Department of Toxicology, Anhui Medical University, Hefei, Anhui, China; 4 Department of Obstetrics and Gynecology, Renji Hospital, Shanghai Jiao Tong University School of Medicine, Shanghai, China; 5 Center for Reproductive Medicine, Renji Hospital, Shanghai Jiao Tong University School of Medicine, Shanghai, China

## Abstract

Premature ovarian failure (POF) is a heterogeneous disease. Though dozens of candidate genes have been identified for the genetic etiology of POF, it is largely unexplained in majority of patients. Recently, *Wt1*^*+/R394W*^ mice was found to present POF-like phenotype, which indicates that *WT1* might be a plausible candidate gene for non-syndromic POF. The coding region of *WT1* gene was screened in 384 patients with POF and 6 novel variations were identified, including two missense mutations (p. Pro126Ser in exon1 and p. Arg370His in exon7) and four intronic variants (c.647-27C > T, c.647-13G > C, c.647-13G > A in intron1 and c.950 + 14T > C in intron 4). *In vitro* experiments showed that both mutant p. Pro126Ser and p. Arg370His repressed the expression of *Amh* and *Cdh1*, and induced the expression of *Fshr* and *Cyp19* in mRNA level (*P *< 0.05). The expression changes of AMH, FSHR, CYP19 and CDH1 were confirmed by western blot. These genes (*AMH*, *FSHR*, *CYP19* and *CDH1*) are required for granular cells (GCs) proliferation, differentiation and oocyte-GCs interaction. The novel mutant p. P126S and p. R370H in the *WT1* gene potentially impaired *GCs* differentiation and oocyte-GCs interaction, which might result in loss of follicles prematurely. Therefore, *WT1* is a plausible causal gene for POF.

Premature ovarian failure (POF), also known as premature menopause or primary ovarian insufficiency (POI), is a heterogeneous disorder characterized by cessation of normal ovarian function before the age of 40 years. It affects approximately 1% of women under 40 yrs and only 0.1% or less under 30^1^,^2^. Genetic factors are considered to be an important facet of the etiologies for POF. A positive family history has been reported in 12.7% of idiopathic POF cases^3^. In addition, a number of variants in different genes have been identified functionally causative, e.g., *NOBOX*, *PGRMC1*, *BMP15* and *FOXL2*^4^,^5^,^6^,^7^. More recently, the largest cohort of genome-wide association study (GWAS) in 791 patients with POF has discovered a susceptible locus at 8q22.3, however, it was a gene desert region and considered as an important yet undefined long distance regulatory region affecting oogenesis^8^. Therefore, the etiology in major cases remains unclear and the underlying mechanisms are largely unknown.

The Wilms’ tumors gene *WT1* encodes an essential transcription factor, which functions in mammalian urogenital development^9^. It was initially identified as a tumor suppressor associated with Wilms’ tumors (a recessive childhood nephroblastoma)^10^, and the mutation or overexpression of *WT1* may lead to glomerulosclerosis of the kidney, gonadal dysgenesis and leukemia^11^,^12^. In adult reproductive organs, WT1 is expressed in Sertoli cells of the tests and granulosa cells (GCs) of ovarian follicles^13^. In rat, the expression of WT1 in GCs decreased along with the follicular development. *WT1* was speculated as a repressor of genes responsible for GCs proliferation and differentiation^14^. More recently, the *Wt1*^*+/R394W*^ mice (a common *WT1* missense mutation in Denys-Drash syndrome) has been shown to be subfertile due to defect in follicle development. Compared to controls, the *Wt1*^*+/R394W*^mice have smaller ovaries and significantly fewer follicles, which is similar to the characteristics of patients with POF^15^. These findings indicate that the *WT1* gene play a critical role in follicle development and might be a plausible candidate for POF. Therefore, we screened the coding region of *WT1* gene in 384 Chinese women with POF to determine its contribution involved in the etiology of POF.

## Results

### Clinical characteristics of patients and two novel missense mutations identified within gene *WT1*

**Figure 1:**
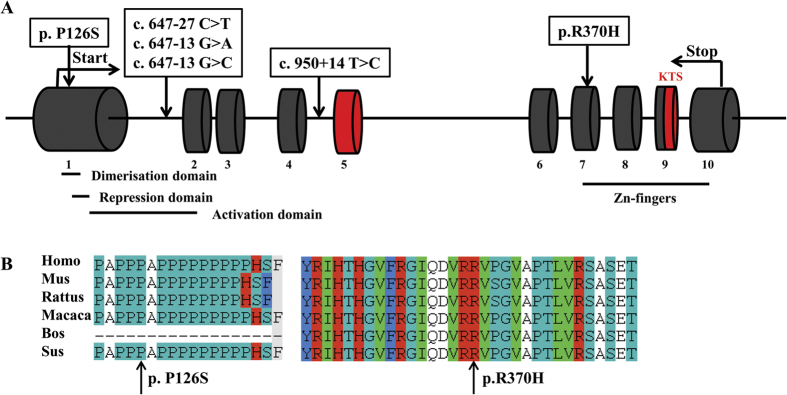
Mutations in *WT1* gene associated with POF and the alternative splices were depicted in red. (**A**) Schematic presentation of *WT1* mutations associated with POF. (**B**) Sequence alignment of WT1 among orthologs with arrow heads indicated.

Clinical characteristics of the 384 subjects were showed in Table 1. Two novel heterozygous missense mutations c.376C > T (p. Pro126Ser, p. P126S) in exon 1 and c.1109G > A (p. Arg370His, p. R370H) in exon 7 were identified in two patients, respectively (Fig. 1A). The two variants were absent in either dbSNP database or 384 control females. The amino acids involved were highly conserved among *WT1* orthologs (Fig. 1B).

The patient carrying p. P126S mutation was 28 years old. She suffered from primary amenorrhea with elevated serum FSH (77.23 IU/L). The other patient with p. R370H developed spontaneous menarche at the age of 16, and experienced irregular menses and subsequent amenorrhea at the age of 31. Trans-vaginal ultrasonography showed normal uterus and small ovaries with severe fibrosis. No other family member of the two carriers had POF or a form of 46, XY disorders of sex development (DSD).

In addition, 10 known single nucleotide polymorphisms (SNPs) and 4 novel intronic variants were found (Table 2, Fig. 1A). The 10 known SNPs included 8 intronic variants (rs2234585, rs1799933, rs5030170, rs5030171, rs2295081, rs5030277, rs192734605 and rs1799937) and 2 synonymous variants (c.330T > C and c.1107G > A). However, the genotype and allelic frequencies of the 10 SNPs between cases and the Asian population showed no significant differences. Of the 4 novel intronic variants, 3 variants were in intron 1 (c.647-27C >T , c.647-13G > A and c.647-13G > C) and 1 was in intron 4 (c.950 + 14T > C). They were not presented in controls.

### Mutant p. P126S and p. R370H impaired the regulations of AMH, FSHR, CYP19 and CDH1 expression

**Figure 2:**
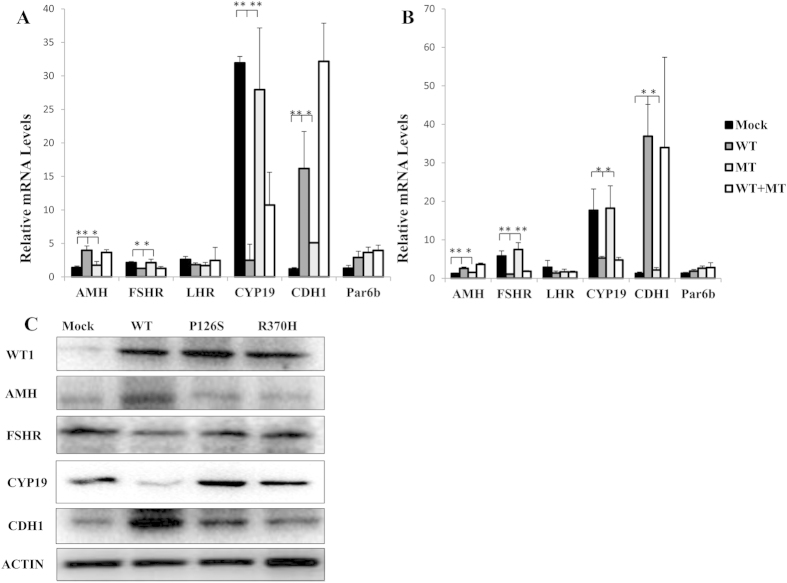
Transfection of empty (Mock), wild-type (WT), or mutant (MT) *Wt1*-expressing adenovirus was performed in differentiated granulosa cells. The potential dominant negative effect of the mutants was assessed by co-transfecting WT expressing adenovirus with MT adenovirus (1:1). The results of p. P126S mutant were showed in (**A**) and p. R370H in (**B**). *P < 0.05, **P < 0.01. (**C**). The GCs were transfected with empty (Mock), wild-type (WT), mutant p. P126S and p. R370H *Wt1*-expressing adenovirus separately. Western blot analyzed nuclear extracts from GCs using antibodys against ACTIN, WT1, AMH, FSHR, CYP19 and CDH1, respectively. Blot images were cropped for comparison.

To explore the effect of mutant p. P126S and p. R370H on transcription, adenovirus vectors containing *Wt1* gene were generated and transfected to differentiated GCs from three-weeks-mouse. Transcription level of down-stream genes including *Amh*, *Fshr*, *Lhr*, *Cyp19*, *Cdh1* and *Par6b* were detected by qRT-PCR. As shown in Fig. 2A,B, in the wild type (WT) *Wt1*-overexpressing differentiated GCs, the expression of *Amh*, *Cdh1*, and *Par6b* was up-regulated while *Fshr*, *Lhr*, and *Cyp19* down-regulated by *Wt1*, which was consistent with previous studies (15). In both p. P126S and p. R370H transfected GCs, the expression of *Amh* and *Cdh1* was decreased and expression of *Fshr* and *Cyp19* was increased (P < 0.05). Given the heterozygous status of two patients carrying p. P126S and p. R370H, dominant negative effect of the mutant over WT protein was further investigated. Co-transfection of WT with mutant p. P127S and p. R387H did not interfere with the transcription of WT protein (Fig. 2A,B), which did not support the dominant negative effect.

Western blot was performed to compare the expression of AMH, FSHR, CYP19 and CDH1 between WT *Wt1* and mutant *Wt1*-overexpressed GCs. As shown in Fig. 2C, the expression of FSHR and CYP19 were repressed in WT *Wt1*-transfected GCs; whereas higher expression was observed in GCs transfected either with p. P126S or with p. R370H. The level of AMH and CDH1 was significantly lower in GCs overexpressed either with p. P126S or with p. R370H compared with WT *Wt1*. These findings confirmed the impaired regulations of mutant p. P126S and p. R370H on AMH, FSHR, CYP19 and CDH1.

## Discussion

The p. P126S mutant located in the proline/glutamine rich DNA-binding domain and p. R370H was adjacent to the four zinc-finger domains. The zinc-finger domains are crucial for *WT1* to acquire the ability to interact with transcription and splice factors^16^. In mice, *Wt1* plays a key role in follicular development regulating these genes associated with GCs proliferation and differentiation, e.g. *Amh*, *Fshr*, *Lhr* and *Cyp19*, and GCs polarity, e.g. *Cdh1* and *Par6b*^15^. Dysregulation of these genes could result in immature differentiation of GCs and impaired establishment of polarity, and then aberrant follicular development.

FSHR is detected in GCs from primary follicle and remains present throughout folliculogenesis^17^. CYP19, encoding aromatase cytochrome P450 (P450arom), is expressed in GCs of large follicles and corpus luteum^18^. FSHR and CYP19 trigger the proliferation and differentiation of GCs, which is important for normal follicular growth^19^,^20^,^21^. As a transcriptional repressor of FSHR and CYP19, WT1 inhibits the premature proliferation and differentiation of GCs. In this study, mutant p. P126S and p. R370H induced FSHR and CYP19 expression, resulting in possible impaired estrogen synthesis, immature proliferation and differentiation of GCs.

AMH is expressed in primary follicles and preantral/small antral follicles^22^,^23^. AMH inhibits the recruitment of primordial follicles and decreases the responsiveness of growing follicles to FSH^24^. Our results revealed that mutant p. P126S and p. R370H repressed the expression of AMH, which would result in increased sensitivity to FSH. Therefore, it was speculated that p. P126S and p. R370H increased the FSH-responsiveness of follicles and promote the activation of primordial follicles.

In the early growing follicle, adherent junctions (AJs) are key adhesion complex^25^. Cadherins and nectins are two important molecules associated with AJs. The most well- characterized cadherins are E-cadherin (official symbol CDH1) and N-cadherin (official symbol CDH2)^26^. CDH1 is a calcium-dependent cell adhesion protein localized at the adherent junctions and serves as a marker for epithelial differentiation^27^. It is essential for maintaining the cuboidal shape of GCs, providing a means of rotating the orientation of mitotic axes and enabling multi-layering of GCs^28^. In this study, mutant p. P126S and p. R370H repressed the expression of CDH1 protein. It is speculated that declined *CDH1* levels could not maintain the oocyte-GC interaction and the loss of granulosa cell adhesion would result in follicular atresia^25^,^29^.

To our knowledge, this is the first study to explore the role of the *WT1* gene in human POF. Two novel heterozygous mutations p. P126S and p. R370H were identified, and resulted in impaired transcription on downstream genes, including *AMH*, *FSHR*, *CYP19* and *CDH1*. They could interfere with the proliferation and differentiation of GCs and disrupt the oocyte-GC interaction. Further studies focusing on the signaling pathways associated with *WT1* in ovary are warranted to reveal the mechanism involved in POF.

## Methods

### Ethics statement

The study procedures were approved by the Institutional Review Board of Reproductive Medicine of Shandong University. All the methods described here were carried out in accordance with the guidelines and regulations approved by the Institutional Review Board of Reproductive Medicine of Shandong University.

### Patients

A total of 384 unrelated Han Chinese women with POF were recruited. Inclusion criteria consisted of primary amenorrhea (PA) or secondary amenorrhea (SA) for at least 6 months before 40 years of age and at least two serum FSH measurements exceeding 40 IU/L. Women with known karyotype abnormalities, previous chemotherapy or radiotherapy, ovarian surgery, or autoimmune diseases were excluded. Patients accompanying with somatic anomalies were excluded, particularly any reported as associated with pleiotropic Mendelian disorders (e.g., blepharophimosis-ptosis-epicanthus inversus syndrome, neurosensory deafness and cerebellar ataxia). Three hundred and eighty-four Han Chinese women with regular menses and normal hormone level were included as controls. Written informed consent was obtained from each subject.

### Mutation Screening of *WT1* Gene

Genomic DNA was extracted from peripheral blood samples with QIAamp DNA mini kit (QIAGEN, Hilden, Germany) according to the manufacturer’s protocol. All 10 exons and exon-intron boundaries of the *WT1* gene (NM_000378.4) were amplified using polymerase chain reaction (PCR). All *WT1* primers and PCR conditions are presented in Supplementary Table S1. The PCR product was first analyzed by agarose gel electrophoresis and then sequenced on an automated sequencer (ABI 3730 × 1 DNA Analyzer; Applied Biosystems, Foster City, CA). The novel sequence variants were confirmed by three independent PCR runs, followed by sequencing in both forward and reverse directions. Nomenclature of variants identified was established according to Human Genome Variant Society (HGVS, www.hgvs.org/mutnomen).

### Adenovirus vectors construction

The mutant c.376C > T (p. P126S) and c.1109G > A (p. R370H), which were found in patients with POF are homologous to c. 187C > T (p. P127S) and c. 905G > A (p. R387H) in mouse, respectively. *Wt1* expression vectors containing c. 187C > T and c. 905G > A were generated by site-directed mutagenesis (QuikChange Lightning Site-Directed Mutagenesis Kit; Stratagene, LaJolla, CA) with WT mouse *Wt1* expression vector as a template. A recombinant adenovirus plasmid was generated by GV adenovirus vector system, pHelper 1.0 vector and pHelper 2.0 vector (Genechem, Shanghai, CH) and then packaged by AdMax system (Microbix, CA). A high titer (1 × 1011 PFU/ml) of adenovirus was obtained by two rounds of amplification and purified.

### Granulosa cells isolation and quantitative RT-PCR analysis

Female mouse at the age of three weeks were injected with 5 IU pregnant mare serum gonadotropin (PMSG), and the ovaries were harvested at 46–48 h after PMSG injection. GCs from pre-ovulatory follicles were collected and cultured overnight for adhesion. Twenty-four hours after adenovirus vectors containing *Wt1* gene infected, GCs were harvested and RNA was extracted. After reverse transcription (Takara, Japan), quantitative real-time PCR (qRT-PCR) was performed on the Roche Lightcycle 480 using specific primers (listed in Supplementary Table S2) with PrimeScript RT Master Mix (Takara). The mRNA levels were normalized against glyceraldehyde-3-phosphate dehydrogenase (GAPDH) and analyzed using the comparative cycle threshold method.

### Western blot

The nuclear extracts from infected GCs of empty (Mock), wild-type (WT), mutant p. P126S and p. R370H *Wt1*-expressiong adenovirus separately were added to 5× sodium dodecyl sulfate (SDS) sample buffer and heated at 95 °C for 5 minutes to denature the proteins. The nuclear extracts were then separated on 10% or 6% SDS-PAGE gels and transferred to polyvinylidene difluoride membranes. The membranes were blocked, incubated with primary antibodies against ACTIN (catalog # 60008-1-lg, Proteintech, CH), WT1 (catalog # sc-192, Santa Cruz, USA), AMH (catalo # sc-166752, Santa Cruz), FSHR (catalog # 22665-1-AP, Proteintech), CYP19 (catalog # 16554-1-AP, Proteintech ) and CDH1 (catalog # 610182, BD Biosciences, USA ) overnight at 4 °C, washed, and then incubated with horseradish peroxidase-conjugated secondary antibodies. Chemiluminescent detection was performed using immobilon western chemiluminescent HRP substrate (Millipore Corporation, Billerica, USA).

### Statistical analysis

Results of qRT-PCR were expressed by bar graphs plotted in MS Excel and represented the Mean ± S.D. of 3 independent experiments, each performed in triplicate. Experiments were repeated at least tree times. The data were performed by chi-squared test and one-way ANOVA. Statistical differences were considered significant when *P *< 0.05.

## Additional Information

**How to cite this article**: Wang, H. *et al.* Novel *WT1* Missense Mutations in Han Chinese Women with Premature Ovarian Failure. *Sci. Rep.* **5**, 13983; doi: 10.1038/srep13983 (2015).

## Supplementary Material

Supplementary Information

## Figures and Tables

**Table 1:** Clinical characteristic of 384 Han Chinese women with POF.

Characteristics	Mean ± S.D./N (%)
Age at diagnosis (yr)	29.81 ± 5.27
Age at menarche (yr)^a^	14.43 ± 1.72
Age of amenorrhea (yr)^a^	25.72 ± 5.77
FSH (IU/L)	72.98 ± 32.66
E_2_ (pmol/L)	27.76 ± 23.59
Family history^b^	21 (5.5%)
Parental consanguinity	3 (0.8%)

^a^Refer to patients with secondary amenorrhea.

^b^A positive family history was considered if another first or second degree female family member had POF or early menopause (menopause before 45 years old).

**Table 2:** Known single nucleotide polymorphisms (SNPs) and novel intron variants identified in patients with POF.

Variation	Location	Amino acid variation	dpSNP ID	Genotype frequency (%)		Allele frequency (%)		*P*-value^c^	Reference^d^
				POF	Asian Population^a^/controls^b^	POF	Asian Population^a^/controls^b^		
c.330T > C	Exon 1	Synonymous	rs1799925	TT(46.0)	NA	T(68.5)	T(67.5)	>0.05	f, g
				TC(44.9)	NA	C(31.5)	C(32.5)		
				CC(9.1)	NA				
c.647-27C > T	Intron 1		Novel	CC(99.7)	CC(100)	C(99.9)	C(100)	>0.05	f, g
				TC(0.3)	TC(0)	T(0.1)	T(0)		
				TT(0)	TT(0)				
c.647-13G > A	Intron1		Novel	GG(99.7)	GG(100)	G(99.9)	G(100)	>0.05	f, g
				GA(0.3)	GA(0)	A(0.1)	A(0)		
				AA(0)	AA(0)				
c.647-13G > C	Intron1		Novel	GG(99.7)	GG(100)	G(99.9)	G(100)	>0.05	f, g
				GC(0.3)	GC(0)	C(0.1)	C(0)		
				CC(0)	CC(0)				
c.770-57T > C	Intron 2		rs2234585	TT(45.3)	NA	T(69.8)	T(65.8)	>0.05	f, g
				TC(49.0)	NA	C(30.2)	C(34.2)		
				CC(5.7)	NA				
c.872 + 16G > A	Intron 3		rs1799933	GG(97.4)	GG(95.6)	G(98.5)	G(97.8)	>0.05	f, g
				GA(2.2)	GA(4.4)	A(1.5)	A(2.2)		
				AA(0.4)	AA(0)				
c.872 + 82T > G	Intron 3		rs5030170	TT(50.9)	NA	T(71.5)	T(65.8)	>0.05	f, g
				TG(41.2)	NA	G(28.5)	G(34.2)		
				GG(7.9)	NA				
c.872 + 85C > G	Intron3		rs5030171	CC(45.6)	NA	C(67.1)	C(65.8)	>0.05	f, g
				CG(43.0)	NA	G(32.9)	G(34.2)		
				GG(11.4)	NA				
c.950 + 14T > C	Intron 4		Novel	TT(99.7)	TT(100)	T(99.9)	T(100)	>0.05	f, g
				TC(0.3)	TC(0)	C(0.1)	C(0)		
				CC(0)	CC (0)				
c.950 + 85G > A	Intron 4		rs2295081	GG(50)	GG(52.2)	G(71.7)	G(71.7)	>0.05	f, g
				AG(43.4)	AG(39.1)	A(28.3)	A(28.3)		
				AA(6.6)	AA(8.7)				
c.1107G > A	Exon 7	Synonymous	rs16754	GG(49.6)	GG(63.4)	G(71.4)	G(76.8)	>0.05	f, g
				AG(43.6)	AG(26.8)	A(28.6)	A(23.2)		
				AA(6.8)	AA(9.8)				
c.1249 + 134A > T	Intron 7		rs5030277	AA(51.3)	AA(56.1)	A(71.1)	A(73.2)	>0.05	f, g
				AT(39.5)	AT(34.1)	T(28.9)	T(26.8)		
				TT(9.2)	TT(9.8)				
c.1432 + 42T > A	Intron 9		rs192734605	TT(99.2)	NA	T(99.6)	T(99.0)	>0.05	f, g
				TA(0.8)	NA	A(0.4)	A(1.0)		
				AA(0)	NA				
c.1433-49 C > T	Intron 9		rs1799937	CC(56.0)	CC(63.4)	C(74.5)	C(76.8)	>0.05	f, g
				CT(37.0)	CT(26.8)	T(25.5)	T(23.2)		
				TT(7.0)	TT(9.8)				

NA: Not available.

^a^Data from http://www.ncbi.nlm.nih.gov/SNP/.

^b^The SNPs were compared with Asian population and the novel variants were compared with the controls.

^c^Comparison of the allele frequency between patients with POF and Asian population/controls.

^d^f = Ensembl accession number ENST00000332351. g =  Ensembl accession number ENSP00000331327.

## References

[b1] Coulam C. B., Adamson S. C. & Annegers J. F. Incidence of premature ovarian failure. Obstetrics and gynecology 67, 604–606 (1986). 3960433

[b2] Goswami D. & Conway G. S. Premature ovarian failure. Hum Reprod Update 11, 391–410, 10.1093/humupd/dmi012 (2005). 15919682

[b3] van Kasteren Y. M. *et al.* Familial idiopathic premature ovarian failure: an overrated and underestimated genetic disease? Hum Reprod 14, 2455–2459 (1999). 10527968 10.1093/humrep/14.10.2455

[b4] Kuo F. T., Bentsi-Barnes I. K., Barlow G. M. & Pisarska M. D. Mutant Forkhead L2 (FOXL2) proteins associated with premature ovarian failure (POF) dimerize with wild-type FOXL2, leading to altered regulation of genes associated with granulosa cell differentiation. Endocrinology 152, 3917–3929, 10.1210/en.2010-0989 (2011). 21862621 PMC3176639

[b5] Mansouri M. R. *et al.* Alterations in the expression, structure and function of progesterone receptor membrane component-1 (PGRMC1) in premature ovarian failure. Human molecular genetics 17, 3776–3783, 10.1093/hmg/ddn274 (2008). 18782852 PMC2722898

[b6] Qin Y. *et al.* NOBOX homeobox mutation causes premature ovarian failure. American journal of human genetics 81, 576–581, 10.1086/519496 (2007). 17701902 PMC1950834

[b7] Rossetti R. *et al.* BMP15 mutations associated with primary ovarian insufficiency cause a defective production of bioactive protein. Human mutation 30, 804–810, 10.1002/humu.20961 (2009). 19263482 PMC2677132

[b8] Qin Y. *et al.* Association of 8q22.3 locus in Chinese Han with idiopathic premature ovarian failure (POF). Human molecular genetics 21, 430–436, 10.1093/hmg/ddr462 (2012). 21989058

[b9] Kreidberg J. A. *et al.* WT-1 is required for early kidney development. Cell 74, 679–691 (1993). 8395349 10.1016/0092-8674(93)90515-r

[b10] Gessler M. *et al.* Homozygous deletion in Wilms tumours of a zinc-finger gene identified by chromosome jumping. Nature 343, 774–778, 10.1038/343774a0 (1990). 2154702

[b11] Hohenstein P. & Hastie N. D. The many facets of the Wilms’ tumour gene, WT1. Human molecular genetics 15 Spec No 2, R196–201, 10.1093/hmg/ddl196 (2006). 16987884

[b12] Rosenfeld C., Cheever M. A. & Gaiger A. WT1 in acute leukemia, chronic myelogenous leukemia and myelodysplastic syndrome: therapeutic potential of WT1 targeted therapies. Leukemia 17, 1301–1312, 10.1038/sj.leu.2402988 (2003). 12835718

[b13] Buckler A. J., Pelletier J., Haber D. A., Glaser T. & Housman D. E. Isolation, characterization, and expression of the murine Wilms’ tumor gene (WT1) during kidney development. Mol Cell Biol 11, 1707–1712 (1991). 1671709 10.1128/mcb.11.3.1707-1712.1991 PMC369476

[b14] Hsu S. Y. *et al.* Wilms’ tumor protein WT1 as an ovarian transcription factor: decreases in expression during follicle development and repression of inhibin-alpha gene promoter. Mol Endocrinol 9, 1356–1366, 10.1210/mend.9.10.8544844 (1995). 8544844

[b15] Gao F. *et al.* Wt1 functions in ovarian follicle development by regulating granulosa cell differentiation. Human molecular genetics 23, 333–341, 10.1093/hmg/ddt423 (2014). 24009315

[b16] Ladomery M., Sommerville J., Woolner S., Slight J. & Hastie N. Expression in Xenopus oocytes shows that WT1 binds transcripts *in vivo*, with a central role for zinc finger one. Journal of cell science 116, 1539–1549 (2003). 12640038 10.1242/jcs.00324

[b17] Oktay K., Briggs D. & Gosden R. G. Ontogeny of follicle-stimulating hormone receptor gene expression in isolated human ovarian follicles. The Journal of clinical endocrinology and metabolism 82, 3748–3751, 10.1210/jcem.82.11.4346 (1997). 9360535

[b18] Suzuki T. *et al.* Temporal and spatial localization of steroidogenic enzymes in premenopausal human ovaries: *in situ* hybridization and immunohistochemical study. Molecular and cellular endocrinology 97, 135–143 (1993). 8143896 10.1016/0303-7207(93)90220-e

[b19] Kempisty B. *et al.* Association between the expression of LHR, FSHR and CYP19 genes, cellular distribution of encoded proteins and proliferation of porcine granulosa cells in real-time. Journal of biological regulators and homeostatic agents 28, 419–431 (2014). 25316123

[b20] Nelson D. R. *et al.* The P450 superfamily: update on new sequences, gene mapping, accession numbers, early trivial names of enzymes, and nomenclature. DNA and cell biology 12, 1–51 (1993). 7678494 10.1089/dna.1993.12.1

[b21] Robker R. L. & Richards J. S. Hormone-induced proliferation and differentiation of granulosa cells: a coordinated balance of the cell cycle regulators cyclin D2 and p27Kip1. Mol Endocrinol 12, 924–940, 10.1210/mend.12.7.0138 (1998). 9658398

[b22] Munsterberg A. & Lovell-Badge R. Expression of the mouse anti-mullerian hormone gene suggests a role in both male and female sexual differentiation. Development 113, 613–624 (1991). 1782869 10.1242/dev.113.2.613

[b23] Taketo T., Saeed J., Manganaro T., Takahashi M. & Donahoe P. K. Mullerian inhibiting substance production associated with loss of oocytes and testicular differentiation in the transplanted mouse XX gonadal primordium. Biol Reprod 49, 13–23 (1993). 8353178 10.1095/biolreprod49.1.13

[b24] Durlinger A. L., Visser J. A. & Themmen A. P. Regulation of ovarian function: the role of anti-Mullerian hormone. Reproduction 124, 601–609 (2002). 12416998 10.1530/rep.0.1240601

[b25] Mora J. M. *et al.* Characterization and significance of adhesion and junction-related proteins in mouse ovarian follicles. Biol Reprod 86, 153, 151–114, 10.1095/biolreprod.111.096156 (2012). 22321830

[b26] Halbleib J. M. & Nelson W. J. Cadherins in development: cell adhesion, sorting, and tissue morphogenesis. Genes Dev 20, 3199–3214, 10.1101/gad.1486806 (2006). 17158740

[b27] Takeichi M. Cadherin cell adhesion receptors as a morphogenetic regulator. Science 251, 1451–1455 (1991). 2006419 10.1126/science.2006419

[b28] Da Silva-Buttkus P. *et al.* Effect of cell shape and packing density on granulosa cell proliferation and formation of multiple layers during early follicle development in the ovary. Journal of cell science 121, 3890–3900, 10.1242/jcs.036400 (2008). 19001500

[b29] Ryan P. L., Valentine A. F. & Bagnell C. A. Expression of epithelial cadherin in the developing and adult pig ovary. Biol Reprod 55, 1091–1097 (1996). 8902222 10.1095/biolreprod55.5.1091

