# Supplementary material for: Novel WT1 Missense Mutations in Han Chinese Women with Premature Ovarian Failure
Source: Sci Rep. 2015 Sep 11;5:13983. doi: 10.1038/srep13983 (PMC4566091; doi:10.1038/srep13983)
Supplement: Supplementary Information [file srep13983-s1.pdf]

Novel *WT1* Missense Mutations in Han Chinese Women with Premature Ovarian Failure

Huidan Wang [1], Guangyu Li [1], Jun Zhang [2, 3], Fei Gao [2], Weiping Li [4], Yingying Qin [1,*], Zi-Jiang Chen [1,5,*]

Supplementary information:

Supplementary Table S1 Primers used for amplification exons of the *WT1* gene

Exon	Primer sequence	Tm (ºC)	Length(bp)
Exon 1	F1: GCCGGCCCCTCTTATTTGAGCT<br>R1: GCTGCGGTCAAAAGGGGTAGGA	66	979
Exon 2	F2: AAAGTCCTGGAGGCTTGTGG<br>R2: GGAGGGAGACCCAGTCTTGTC	59	449
Exon 3	F3: AGGCTCAGGATCTCGTGTCT<br>R3: GGTAAATTCCTCCCAGTAAAG	55	497
Exon 4	F4: CTGTGCAGAGATCAGTGGGAT<br>R4: GTGTCCTCAATATTCCTTGTTCC	55	485
Exon 5	F5: TCTTTGCAGCCTCCGGTGGT<br>R5: ATGCTACCCTGATTACCCACGTC	62	350
Exon 6	F6: GAGCGAGACCTAGACCGTGAC<br>R6: ACAGCCGCCTTATCAGACCC	62	528
Exon 7	F7: AGGTTTCATCTCAACAGCCACT<br>R7: ACCACTCTGCTCTGCCTTTCT	59	631
Exon 8	F8: GCTCCCATTCATTTGTAACTT<br>R8: ATCACCCTCATTCTTTGCTG	55	536
Exon 9	F9: GTTCCCAAGACAGAAGCAGG<br>R9: ACAGTAGGGACCTGGCTTATCT	57	581

Exon 10	F10: GACTCATTCTTTATCTCCCACTTTC<br>R10: CATGATCAGCTATGGCTCTTCTTAC	59	684

Supplementary Table S2 Primers used for qRT-PCR

Gene	Primer sequence
GAPDH	F: TGATGACATCAAGAAGGTGGTGAAG<br>R: TCCTTGGAGGCCATGTAGGCCAT
Wt1	F: CAAGGACTGCGAGAGAAGGTTT<br>R: TGGTGTGGGTCTTCAGATGGT
AMH	F: CCACACCTCTCTCCACTGGTA<br>R: GGCACAAAGGTTCAGGGGG
FSHR	F: CCTTGCTCCTGGTCTCCTTG<br>R: CTCGGTCACCTTGCTATCTTG
LHR	F: CTCGCCCGACTATCTCTCAC<br>R: ACGACCTCATTAAGTCCCCTG
CYP19	F: AACCCCATGCAGTATAATGTCAC<br>R: AGGACCTGGTATTGAAGACGAG
CDH1	F: CGAGAGAGTTACCCTACATA<br>R: GTGTTGGGGGCATCATCATC
Par6b	F: GGAGCTGAGTTTCGTCGGTTT<br>R: CGTAGCCCACCAACACGTC
